# Case series: Noninfectious myositis of temporal muscle: a report of 2 cases

**DOI:** 10.1097/MD.0000000000034100

**Published:** 2023-06-23

**Authors:** Mi-Sun Kong, Kyung-Hoe Huh, Hong-Seop Kho

**Affiliations:** a Department of Oral Medicine and Oral Diagnosis, School of Dentistry and Dental Research Institute, Seoul National University, Jongno-gu, Seoul, South Korea (ROK); b Department of Oral and Maxillofacial Radiology, School of Dentistry and Dental Research Institute, Seoul National University, Jongno-gu, Seoul, South Korea (ROK); c Institute on Aging, Seoul National University, Seoul, South Korea (ROK).

**Keywords:** case report, myositis, temporal muscle

## Abstract

Noninfectious myositis (NIM) of the masticatory muscles is uncommon local myalgia disorder persisted by a centrally-mediated neurogenic mechanism. Due to the rarity of this condition and the lack of appropriate data regarding it, diagnosing this pathology when it affects the temporal muscle (TM) is challenging.

**Case presentation:**

Clinical signs and symptoms, diagnostic process, and treatment outcome of 2 rare cases of NIM of the TM were presented. The signs and symptoms of the patients were not pathognomonic. There were restrictions on the mouth opening and lateral excursion of the mandible. The duration of the symptoms may not be chronic. The findings of clinical evaluation may indicate the diagnosis of anterior disc displacement (DD) without reduction of the temporomandibular joint (TMJ) and/or local myalgia. Swelling of the involved muscle could be evident and identified on palpation depending on the involved site of myositis. The axial T2-weighted magnetic resonance (MR) imaging was important for the accurate diagnosis of this rare condition. Application of non-surgical conservative treatment modalities such as administration of non-steroidal anti-inflammatory analgesics for a sufficient period of time, control of oral parafunctional habits, and jaw exercises were effective for the management of NIM of the TM.

**Conclusion:**

A thorough clinical examination and MR imaging including the axial T2-weighted view are required for accurate diagnosis and effective management of NIM of the TM.

## 1. Introduction

Most myositis in the masticatory muscles is caused by infection and is often accompanied by cellulitis.^[1,2]^ However, rare noninfectious myositis (NIM) may develop in the masticatory muscles. The primary mechanism has been proposed to be central sensitization brought on by persistent local myalgia and the ensuing neurogenic inflammation.^[3]^ As a result, NIM of the masticatory muscles is called centrally-mediated myalgia or persistent orofacial muscle pain.^[3,4]^

In patients with NIM of the masticatory muscles, typical inflammatory signs such as edema, redness, and fever are not evident.^[3]^ The patient complaints may not be significantly different from those of common temporomandibular disorders.^[5]^ The absence of pathognomonic signs and symptoms in patients with NIM makes it challenging for clinicians to make an accurate diagnosis and efficient management. Therefore, using advanced imaging modalities could be important for accurate diagnosis of NIM of the masticatory muscles.^[5,6]^ Rare cases of NIM of the lateral pterygoid muscle (LPM) diagnosed by enhanced computed tomography or magnetic resonance (MR) imaging have been reported.^[5]^ Some important clinical clues to help the diagnosis of NIM of the LPM has been suggested.^[5]^ Here, we report 2 cases of patients with jaw pain and limited mouth opening, which were finally identified as uncommon NIM of the temporal muscle (TM).

## 2. Case presentation

Two cases were from patients who visited one doctor (HSK) at the temporomandibular joint (TMJ)-Orofacial pain clinic, Department of Oral Medicine, Seoul National University Dental Hospital from January 2011 to December 2020. The research protocol was reviewed in compliance with the Helsinki Declaration and approved by the Institutional Review Board of the University Hospital (#ERI23001). The Institutional Review Board authorized an exemption of informed consent from the subjects. All procedures were carried out in accordance with relevant guidelines and regulations.

### 2.1. Case 1

A 26-year-old woman visited with left jaw pain and limited mouth opening that had increased 8 days prior. She visited a local dental clinic 7 days prior and was prescribed non-steroidal anti-inflammatory analgesic drugs (NSAIDs) and muscle relaxants without help. Her symptoms increased 3 days before the visit. She reported that she had clicking on the left TMJ several months prior and had left jaw pain and limited mouth opening 3 months prior. At that time, her symptoms had decreased some after taking NSAIDs for 7 days, after which she still had persistent mild pain.

She was unable to open her mouth more than 23 mm. She reported pain in her left jaw on protrusive and right lateral excursive movements. She experienced a clicking sound in her left TMJ when she moved her mandible laterally. She had pain on palpation of the left TMJ capsule and left deep masseter area. There was no swelling or local heat on the face. She admitted to having a clenching habit. On panoramic and transcranial radiographs, no distinctive bony changes were detected in both TMJ areas.

She was clinically diagnosed with anterior disc displacement (DD) without reduction of left TMJ, arthralgia of left TMJ, and local myalgia of left masseter muscle. She was given aceclofenac 100 mg bid for 2 weeks. She was also educated about soft diet and parafunctional habit control such as clenching. Her symptoms somewhat subsided 2 weeks later, but TMJ MR imaging was done because of her mouth opening, which was about 25 mm.

**Figure 1.**
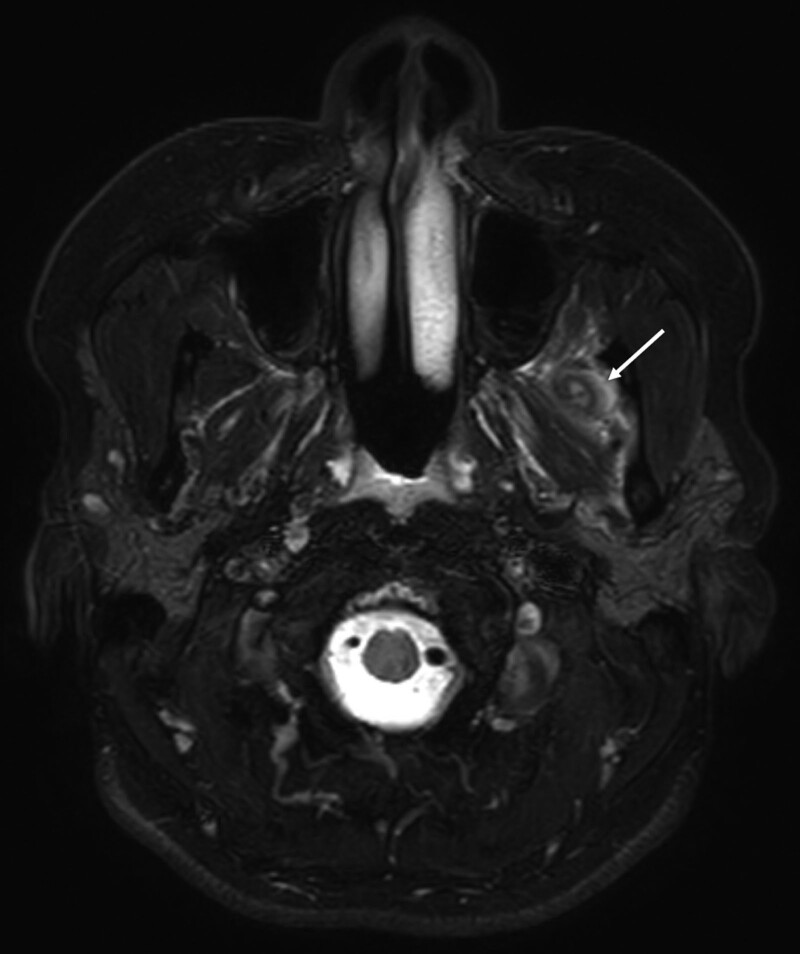
Fat-suppressed axial T2-weighted MR image shows a heterogenous hyperintense T2 signal lesion in the left temporal muscle near the medial aspect of the left mandibular ramus and the coronoid process, indicating myositis of the left temporal muscle. MR = magnetic resonance.

MR imaging showed that both TMJs had partial anterolateral DD in the closed-mouth view. Both displaced discs did not show a reduced state in the restricted open-mouth view. In the fat-suppressed axial T2-weighted image, a lesion showing a hyperintense T2 signal was found in the TM near the medial aspect of the left mandibular ramus and the coronoid process (Fig. 1). The patient was diagnosed with NIM of the left TM. An exercise was instructed to gradually increase the mouth opening within a pain-free range. Two weeks after, her mouth opening was 48 mm and she reported that a slight stiffness was felt on the left TMJ at the maximum opening. No tenderness was felt on both TMJ capsular or masticatory muscle areas. When she returned to the clinic 3 months later, the mouth opening was comfortably increased to 50 mm.

### 2.2. Case 2

A 35-year-old man visited with pain and swelling in the right temple area and limited mouth opening that had begun 1 week prior. He was referred to the Department of Infectious Diseases in one tertiary medical hospital after being prescribed antibiotics and NSAIDs at the internal medicine clinic for 3 days without help. After being determined to have a minimal risk of infection, he was recommended to our clinic.

He was unable to open his mouth more than 35 mm due to both sides of jaw pain, which was more severe on the right. Deflection of the mandible to the right side was observed on mouth opening. He exhibited restricted lateral movement of the mandible bilaterally and complained of excruciating pain on the right side of his jaw during both lateral excursive movements. No TMJ noise was observed. Swelling and severe pain on palpation were found in the right middle and posterior TM area. However, no local heat was felt in that area. There was no pain on palpation of both TMJ capsular and masseter muscle areas. On plain radiographs, no specific bony changes were identified in both TMJ areas. No site could be presumed to be an infection origin in the oral and maxillofacial area.

He was clinically diagnosed with NIM of the right TM. Although bulging of the temporal artery was not observed, temporal arteritis was considered a differential diagnosis. Complete blood counts with erythrocyte sedimentation rate and TMJ MR imaging were carried out.

**Figure 2.**
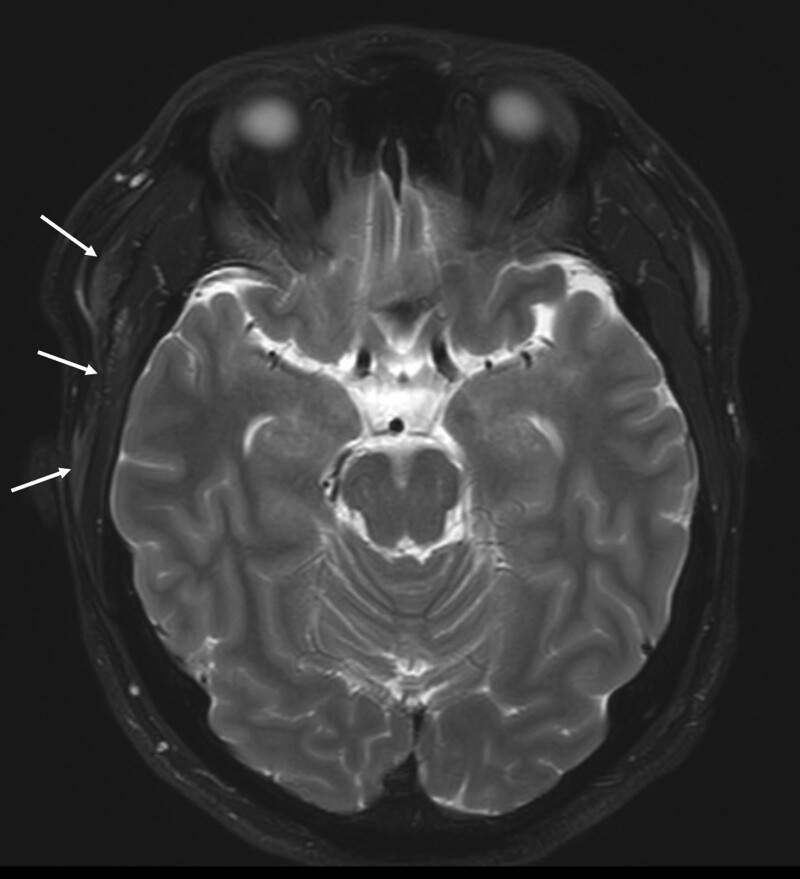
Fat-suppressed axial T2-weighted MR image reveals swelling of right temporal area and a hyperintense T2 signal lesion in the right temporal muscle, indicating myositis of the right temporal muscle. MR = magnetic resonance.

MR imaging revealed that both TMJ discs were in the normal position in both open- and closed-mouth views. In the fat-suppressed axial T2-weighted image, a lesion revealing a hyperintense T2 signal was found in the right TM (Fig. 2). The results of complete blood counts with erythrocyte sedimentation rate were within the normal range. The patient was diagnosed with NIM of the right TM. He was prescribed naproxen 500 mg bid for 2 weeks. Two weeks later, he noted that the swelling and pain had subsided, and his mouth had opened to a 40 mm. He has been prescribed the NSAIDs for 2 weeks again. An exercise was instructed to gradually increase the mouth opening. His symptoms were almost relieved 2 weeks later.

## 3. Discussion

Our cases reveal the importance of MR imaging, particularly the fat-suppressed axial T2-weighted one in the diagnosis of NIM of the TM. Since TMJ MR imaging typically includes the sagittal and coronal views, adding the axial image is strongly advised as a standard practice for patients with temporomandibular disorders.

Based on our cases, the duration of the patient complaint looks not crucial for the development of NIM. In case 1, there was a history of symptoms for several months, but in case 2, there was a history of symptoms for about a week. However, an increase in muscle tension that did not cause obvious level of pain may have existed for a long period of time. In NIM, it has been suggested that typical inflammatory signs are not evident.^[3]^ However, swelling of the involved muscles was found in the LPM^[5]^ and TM by MR imaging. In case 2, swelling could be detected by clinical palpation. However, local heat was not felt, and redness was not found. The tenderness of the muscle area where myositis occurred was evident. However, there may be cases where the muscles are nearly impossible to palpate.^[5]^ In the both cases of the present study, the mouth opening and lateral movements were restricted and jaw pain was noticeable in these movements, though these could not be pathognomonic. Therefore, MR imaging with an axial view is strongly recommended when the results of clinical examination do not suggest a probable diagnosis. This can avoid unnecessary treatments including the administration of antibiotics, injections, and other surgical procedures which may exacerbate the symptoms.^[3]^

Our cases show the importance of administering NSAIDs for a sufficient period of time to decrease inflammation. Control of parafunctional habits might provide additive benefits. Restriction of mandible use within a painless limit in the early phase and an exercise to gradually increase the mouth opening within a pain-free range in the later phase were also helpful. Low dosages of tricyclic antidepressants or cyclobenzaprine have been recommended to help sleep as well as to decrease muscle pain.^[3,7]^ In our cases, it was not prescribed because the symptoms were controlled.

Our cases provide several important information to understand the pathophysiology, diagnosis, and treatment of NIM of the TM. First, the symptoms of the patients were not pathognomonic, and the duration of the symptoms may not be chronic. Second, the results of the clinical evaluation may suggest the diagnosis of DD without reduction and/or local myalgia. Third, swelling of the involved muscle was evident and could be palpated according to the involved muscle area. Fourth, the axial T2-weighted MR imaging was important for accurate diagnosis.

## 4. Conclusions

A thorough clinical examination and MR imaging including the axial T2-weighted view are required for accurate diagnosis and effective management of NIM of the TM. Application of non-surgical conservative treatment is effective for the management of NIM of the TM.

## Author contribution

**Conceptualization:** Mi-Sun Kong, Hong-Seop Kho.

**Investigation:** Mi-Sun Kong, Kyung-Hoe Huh, Hong-Seop Kho.

**Resources:** Kyung-Hoe Huh, Hong-Seop Kho.

**Supervision:** Hong-Seop Kho.

**Writing – original draft:** Mi-Sun Kong, Hong-Seop Kho.

**Writing – review & editing:** Kyung-Hoe Huh, Hong-Seop Kho.
